# An unusual complication of a common endemic disease: clinical and laboratory aspects of patients with brucella epididymoorchitis in the north of Iran

**DOI:** 10.1186/1756-0500-4-286

**Published:** 2011-08-11

**Authors:** Narges Najafi, Roya Ghassemian, Ali R Davoody, Atefe Tayebi

**Affiliations:** 1 Department of infectious disease, North Iranian tropical and infectious disease research center, Mazandran university of medical sciences, Iran

**Keywords:** Brucellosis, Epididymoorchitis, Testicular abscess

## Abstract

**Background:**

Brucella epididymoorchitis(BEO) is a focal complication of human brucellosis and has been reported in 2-20% of patients with brucellosis. Brucellosis is an endemic disease in Iran. The incidence of the disease in this country is 34 per 100 000 per year.

**Methods:**

In a retrospective study, we identified 30 cases of Brucella epididymoorchitis in two teaching hospitals in the north of Iran during 1997-2009.

**Findings:**

Epididymoorchitis occurred in 11.1% of male patients with brucellosis. The average age was 25.5 ± 12.43 years. Pain and scrotal swelling (100%) and fever (96.7%) were the most common symptoms. Different treatment regimens were administered including doxycycline plus rifampin (60%), doxycycline plus rifampin plus aminoglycoside for the first two weeks (36.6%) and doxycycline plus cotrimoxazole(3.4%). Ten percent of the patients did not respond to antibiotic therapy and required surgical drainage or orchiectomy.

**Conclusions:**

In brucellosis endemic areas, clinicians who encounter patients with epididymoorchitis should consider the likelihood of brucellosis. A careful history and physical examination and an immediate laboratory evaluation help to make a correct diagnosis. Generally, classical therapy of brucellosis is adequate for the treatment of epididymoorchitis.

## Introduction

Brucellosis is an endemic zoonotic disease that can involve many organs and tissues. The incidence of brucellosis in developed countries is low, but it occurs sporadically in occupationally exposed groups, including farmers, veterinarians, and laboratory and slaughterhouse workers [1,2]. Brucellosis is an endemic disease in Iran. The incidence of the disease in this country is 34 per 100 000 per year [3]. Brucella epididymoorchitis(BEO) is a focal complication of human brucellosis and has been reported in 2-20% of patients with brucellosis [4-6]. Brucella species were first described as a cause of granulomatous orchitis in humans by hardy in 1928 [7]. Since then, many authors have reported sporadic cases of Brucella orchitis worldwide [5-15]. BEO can cause serious complications such as necrotizing orchitis, and therefore it must be considered in the differential diagnosis of acute epididymoorchitis in endemic areas [4,8-10]. However, genitourinary complications of brucellosis have been documented only rarely in the medical literature [5,8,9]. BEO is relatively uncommon in developed countries because brucellosis has been eradicated generally in animals. Nevertheless, cases have been reported in patients from other countries where the disease is endemic, or in people who have traveled to these areas and have consumed unpasteurized dairy products [2,5-7]. In the present study, we describe the clinical characteristics, treatment, and final outcome of 30 patients with Brucella epididymoorchitis who were hospitalized in two teaching hospitals of Mazandaran University of Medical Science (Razi hospital and Imam Khomeini hospital) during 1997-2009.

## Methods

Among 447 patients confirmed to have brucellosis who were admitted to Razi and Imam Khomeini hospitals in the north of Iran during 1997-2009, thirty patients met the criteria of this study. The diagnosis of brucellosis was made by the isolation of Brucella species from blood culture or by using a standard tube agglutination test, with a titer of ≥ 1:160, the Rose Bengal test (positive flocculation reaction), the Coombs test, with a titer of ≥ 1:160, and the 2ME test, with a titer of ≥ 1:80 for antibodies to Brucella according to standard methods [2]. The results were considered in combination with compatible clinical findings (e.g. orchitis and fever, sweating, arthralgia, hepatomegaly, splenomegaly, and other signs of focal disease). The diagnosis of epididymoorchitis was based on the presence of clinical symptoms of orchitis such as scrotal pain and swelling. Ultrasonography and other diagnostic imaging studies were performed as appropriate for the symptoms of the patients. The patients were assessed initially, on days 14 and 45, and at the end of therapy. Histological finding were described for one patient who required orchiectomy. For each patient a questionnaire was used to abtain information on demographics, clinical findings, laboratory results, treatment, and outcome and the data were analyzed using SPSS descriptive statistical tests.

## Results

A total of 447 cases of brucellosis were diagnosed of which 268 were in males. BEO was diagnosed in 30 of the male patients, hence an incidence of 11.11% in males and 6.70% in all patients with brucellosis was calculated. The mean age of the patients with BEO was 25 ± 12.4 (range, 14-61) years. Sixteen patients (53.3%) were living in rural areas. The most common seasons of occurrence of the illness were spring and summer (13 cases, 43.3% and 12 cases, 40% respectively). Twenty-one patients (70%) had a history of consumption of unpasteurized dairy products, which is the main risk factor for contracting brucellosis, and nine patients (30%) had occupational exposure. Twelve patients (40%) had consumed unpasteurized dairy products as well as being at occupational exposure. One case presented with a relapse of brucellosis, but he had neither consumed unpasteurized dairy products nor had any occupational contact. The most common presentation was scrotal pain and swelling. Scrotal pain and swelling, fever and sweating were the most common symptoms of the disease. Lower urinary tract symptoms were found in 33.3% of the patients (Table 1).

**Table 1:** Specific signs and symptoms in 30 patients with Brucella epididymoorchitis

Findings	Frequency	Percentage
Scrotal pain and swelling	30	100

Fever (temp ≥ 38°c)	29	96.7

Sweating	25	83.3

Anorexia	25	83.3

Weakness	23	76.7

Myalgia	8	26.7

Arthralgia	6	20

Back pain	6	20

Urinary frequency	6	20

Weight loss	4	13.3

Lumbosacral pain	4	13.3

Dysuria	3	10

Splenomegaly	3	10

The erythrocyte sedimentation rate (ESR) was measured in 28 patients. The mean ESR was 36.82 ± 27.92^mm^_/h_. The ESR was abnormal in 57% of cases, and 28.55% of patients had ESR > 50^mm^_/h_. Anemia (Hb < 14^g^/_dl_) was found in 28 patients (93.3%). The platelet counts were normal in all the patients, but leukocytosis (WBC > 12000 mm^3^) was found in 10 cases (33.3%). The level of hepatic transaminase was normal in 28 patients, although it showed a slight increase in two patients. Renal function tests were consistently normal. Urine analysis was also normal in 28 patients (93.3%). Two patients had hematuria and pyuria (6.7%). The Rose Bengal test showed a positive flocculation reaction in all patients. The standard tube agglutination tests and Coombs tests were positive with a titer ≥ 1:160 (1:160-1:2560) in all patients. The 2ME test was also positive in all of the patients (1:80-1:640). Ultrasonography showed unilateral involvement of the epididymis and testis in 26 patients (86.7%), and bilateral involvement was observed in 4 cases (13.3%). Eighteen patients (60%) had orchitis without any change in the epididymis. A testicular abscess was noted in five patients (15%). The mean time that elapsed between admission and initial therapy was 1-7 days (mean: 2.43 ± 1.57 days). All patients received different regimens of orally administered antibiotics, as follows: doxycycline and rifampin in 18 cases (60%), doxycycline and rifampin along with parenteral aminoglycoside for the initial two weeks in 11 cases(36.6%), doxycycline and trimetoprime-sulfamethoxazole in one patients. The average duration of antimicrobial therapy was 45-60 days. Ninty percent of the patients were treated successfully with antimicrobial therapy and experienced rapid regression of symptoms, including defeverscence and diminished scrotal swelling. In this series of patients with BEO, there were no clinically significant differences observed among the different treatment groups. Three patients (10%) failed to respond to medical therapy. Ultrasonography showed a testicular abscess in all three cases, and the three patients underwent surgery. Of these, two patients responded to drainage of the abscess and the other required orchiectomy. The average duration of hospitalization was 7.8 ± 4.5 (range: 3-21) days.

## Discussion

Infections caused by organisms of the genus Brucella can produce orchitis in susceptible mammals, including humans [4]. Brucellosis is a relatively common cause of BEO in some geographic areas, including Iran, where Brucella melitensis is endemic. Only a few case series from Iran that discuss Brucella epididymoorchitis have been published. Rates of epididymoorchitis in cases of human brucellosis have ranged from 2-20% in various reports [2,5,6].

In the current study, epididymoorchitis occurred in 6.70% of all patients and 11.11% of male patients with brucellosis in a 13 year period. In a previous study of 96 patients with epididymoorchitis in Imam Khomeini hospital (1995-1996) BEO was found in 14.6% of cases. Navarro reported BEO in 6% of patients in his study (1988) in Spain [10], but in Guindu-Sevillano's study, 12.8% of cases of epididymoorchitis in Spain were due to Brucella infection [13]. Memish et al. reported BEO in 1.6% of their patients with brucellosis [7]. A seven year study in Turkey (Yurdakal et al.) revealed BEO in 17% of all cases with epididymoorchitis[11]. The rate of epididymoorchitis in our patients was similar to that reported from other studies in endemic areas.

The diagnosis of scrotal disease was based on clinical findings and laboratory data. BEO can be distinguished from other acute nonspecific types of orchitis by various clues, including: gradual onset and longer duration, positive contact history with animals or unpasteurized dairy products, typical undulant fever, and abnormal urologic findings. In most of our patients, as in other studies, fever, scrotal pain and swelling were found, but signs and symptoms of urinary infection were observed in 30% of patients.

In a previous study in Sari Imam Khomeini hospital, signs and symptoms of lower urinary tract infection were found in 28% of patients [12]. In contrast, signs of urinary tract infection were observed in 7% of cases in Spain [9] and 19.2% in Saudi Arabia [5,6]. Khan et al, [14] found lower urinary tract symptoms in 69% of patients, but the other authors describe a characteristic absence of these symptoms in patients with BEO [5,6,13]. According to the different reports of the signs and symptoms of urinary tract infection, we cannot use this finding as a diagnostic criterion for BEO. We must consider BEO in every patient with scrotal swelling and fever, without paying any particular attention to urinary symptoms. The hematological findings are usually non specific and cannot help with the diagnosis of BEO. These disturbances are usually mild. A low level of hemoglobin may be the result of prolonged infection and a moderate elevation in ESR is found in most cases. Most reports describe no changes in urinary sediment, but in our study we noticed a change in two patients, who showed hematuria and pyuria, similar to some patients in the Spanish study [10]. Liver function tests disclosed a mild to moderate increase in the serum level of hepatic transaminase. These abnormalities in liver function tests may be caused by granulomatous Brucella hepatitis, However, when serious liver malfunction is found, intercurrent disease must always be excluded [5]. In our series, 16.7% of patients had mild elevation in liver function tests.

The diagnosis of brucellosis was made by isolation of Brucella species from blood cultures or epididymal aspirates, or by standard tube agglutination tests, revealing a titer of antibodies to Brucella antigen of ≥ 1:160, in addition to compatible clinical findings [5]. Standard urine culture is inadequate for the diagnosis of genitourinary brucellosis.

The presumptive diagnosis of Brucella orchitis can be made by serological testing [4-6,15]. Positive results (titers of antibodies to Brucella species of > 1:160 with the standard tube agglutination test) are common. However, low titers determined by the standard tube agglutination test have been reported, and rarely, some patients with brucellosis have positive blood cultures but negative serological results [4].

In our study, all of the patients had standard tube agglutination titers ≥ 1:160 and those of 2ME > 1:80. Ultrasonography plays an important role in the diagnosis, assessment and management of patients with BEO [4]. Ultrasonography is more useful in excluding the possibility of abscess or tumor than in helping to establish the primary clinical diagnosis [4]. The most notable ultrasonographic finding was an enlarged and heterogeneous epididymis, predominantly the body and tail. Testicular involvement consisted of a diffusely hypoechoic testis or focal intratesticular areas. Thickening of the scrotal wall and tunica albuginea, and moderate hydrocele were also noted occasionally [4,16]. Unilateral epididymoorchitis is the most common genitourinary complication of brucellosis. Infection limited to the testis is rare; the epididymis is usually involved in patients who have acute inflammation [4,5,16]. In the normal epididymis, very few or no vessels are seen on color Doppler sonogram, but the size and number of vessels increase if the epididymis is inflamed. The changes seen in color Doppler images occur sooner than the changes evident on a sonogram [4,5,16].

In our series, 86.4% of patients had unilateral testicular involvement. All of our cases had testicular involvement, and in 40.1% this was accompanied by epididymal involvement. In the study that was conducted in Spain, 91% of patients had unilateral involvement. Epididymitis was found in 41.1% of the cases and changes in echotextures of the testis were detected in 82% of sonograms. The lower number of patients with epididymal involvement reported in our series could be a result of the lack of use of color Doppler sonograms for diagnosis.

The existence of a hypoechoic lesion in the testis sonogram is a sign of testicular abscess formation, and surgery is usually needed in these cases [4,5]. In our study, abscess formation was observed in five patients (16.7%), of which two cases responded to medical treatment. Drainage of the abscess was performed for two cases and orchiectomy in the other.

In a study conducted in Spain, 81% of the patients underwent surgery [5]. In the other studies reviewed the need for surgery was lower. The relatively high frequency of surgery in our patients was probably a result of the fact that the urology center in Imam Komeini hospital is a referral site. Necrotizing orchitis is a rare form of Brucella infection, which must be distinguished from necrotizing involvement arising from other pathogens (eg. Mycobacterium tuberculosis, Salmonella species) [4,5]. In addition, some acute cases may be mistaken for urinary tract infections with Gram-negative pathogens [4]. In brucellosis endemic areas, clinicians who encounter epididymoorchitis should consider the likelihood of brucellosis. A careful history, a meticulous physical examination and a rapid laboratory evaluation will assist the diagnosis. Clinical and serological data are sufficient for diagnosis. Conservative management with a combination of antibiotics is adequate for the management of most cases of Brucella epididymoorchitis.

## Competing interests

The authors declare that they have no competing interests.

## Authors' contributions

NN participated in the design of the study and was involved in drafting the manuscript. RG made contributions to the conception and design of the study and participated in acquisition of data. ARD made contributions to the acquisition of data performed the statistical analysis and helped to draft the manuscript. AT made contributions to the acquisition of data, the analysis and interpretation of the data, and helped to draft the manuscript. All authors have read and approved the final manuscript.
